# Effects of ^28^Si Ions, ^56^Fe Ions, and Protons on the Induction of Murine Acute Myeloid Leukemia and Hepatocellular Carcinoma

**DOI:** 10.1371/journal.pone.0104819

**Published:** 2014-08-15

**Authors:** Michael M. Weil, F. Andrew Ray, Paula C. Genik, Yongjia Yu, Maureen McCarthy, Christina M. Fallgren, Robert L. Ullrich

**Affiliations:** 1 Colorado State University, Fort Collins, Colorado, United States of America; 2 University of Texas Medical Branch, Galveston, Texas, United States of America; National Taiwan University, Taiwan

## Abstract

Estimates of cancer risks posed to space-flight crews by exposure to high atomic number, high-energy (HZE) ions are subject to considerable uncertainty because epidemiological data do not exist for human populations exposed to similar radiation qualities. We assessed the carcinogenic effects of 300 MeV/n ^28^Si or 600 MeV/n ^56^Fe ions in a mouse model for radiation-induced acute myeloid leukemia and hepatocellular carcinoma. C3H/HeNCrl mice were irradiated with 0.1, 0.2, 0.4, or 1 Gy of 300 MeV/n ^28^Si ions, 600 MeV/n ^56^Fe ions or 1 or 2 Gy of protons simulating the 1972 solar particle event (1972SPE) at the NASA Space Radiation Laboratory. Additional mice were irradiated with ^137^Cs gamma rays at doses of 1, 2, or 3 Gy. All groups were followed until they were moribund or reached 800 days of age. We found that ^28^Si or ^56^Fe ions do not appear to be substantially more effective than gamma rays for the induction of acute myeloid leukemia. However, ^28^Si or ^56^Fe ion irradiated mice had a much higher incidence of hepatocellular carcinoma than gamma ray irradiated or proton irradiated mice. These data demonstrate a clear difference in the effects of these HZE ions on the induction of leukemia compared to solid tumors, suggesting potentially different mechanisms of tumorigenesis. Also seen in this study was an increase in metastatic hepatocellular carcinoma in the ^28^Si and ^56^Fe ion irradiated mice compared with those exposed to gamma rays or 1972SPE protons, a finding with important implications for setting radiation exposure limits for space-flight crew members.

## Introduction

The effects of HZE (high Z, high energy) ions found in the space environment on cancer risks to space-flight crews are not well understood because few people have been exposed to space radiation [1]–[6]. In the absence of human exposure data, results from animal studies utilizing simulated space radiation are a key component to risk modeling. The most complete data set is for Harderian gland tumors in the mouse. These data indicate a substantial increase in risks for tumor induction over gamma ray exposure for a number of HZE ions at low doses followed by a bend-over/plateau. To estimate the relative biological effectiveness (RBE) of these ions a linear dose response model over the 0 to 0.5 Gy dose range has been used. A more recent analysis has suggested that a continuously bending dose response provides a better fit to the data [7]. From this analysis it was argued that the continuously bending curve indicated a role for non-targeted effects involved in the pathogenesis of Harderian gland tumors following exposure to both HZE ions and fission spectrum neutrons. Because humans lack Harderian glands, the applicability of these results to human risks has been an important question. With a few exceptions, other investigators have also provided evidence for a substantial increase in risks for the induction of other epithelial tumors following exposure to HZE ions compared to gamma rays with RBE values ranging from approximately 10 to greater than 50 [8].

We have recently described results of a study examining the induction of acute myeloid leukemia (AML) and hepatocellular carcinoma (HCC) in male CBA mice following exposure to either ^137^Cs gamma rays or 1 GeV/n ^56^Fe ions [9]. The RBE for the induction of AML was very low (essentially 1), a result that contributed to the use of a smaller maximum quality factor function for leukemia in the NASA model for space cancer risk [10], [11]. However, the study found a substantial RBE (about 50) for the induction of HCC. Furthermore, the dose response for HCC showed a large increase in the incidence of tumors over the dose range from 0 to 0.4 Gy followed by a bend-over between 0.4 and 1 Gy, similar to that previously described for Harderian gland tumors in mice [12]–[14] and mammary tumors in Sprague Dawley rats [15]. However, the dose response for AML was essentially linear and similar to that observed for gamma rays.

Additional studies have now been conducted in our laboratories extending these investigations to include an examination of the carcinogenic effects of ^137^Cs gamma rays compared to 600 MeV/n ^56^Fe ions, 300MeV/n ^28^Si ions, and protons with energies simulating the 1972 solar particle event (1972SPE). For these studies we used male C3H mice that, as in the case of CBA mice, have been shown to be susceptible to the induction of AML following radiation exposure and to spontaneous HCC. The results of the present study confirm our previous observations with 1 GeV/n ^56^Fe ions of a low RBE for AML and a very high RBE for HCC. No significant differences were observed between the effects of ^137^Cs gamma rays and 1972SPE protons with respect to the induction of either AML or HCC. In addition to the substantial quantitative differences in the effectiveness of HZE ions compared to either gamma rays or protons on the induction of HCC, we also observed qualitative differences as measured by an increase in the frequency of metastases from tumors arising in ^28^Si and ^56^Fe ion irradiated mice compared with those from gamma ray or proton irradiated mice.

## Materials and Methods

### Mice, Irradiations, and Morbidity Monitoring

Eight to ten-week-old male C3H/HeNCrl mice were purchased from Charles River Laboratories and shipped to the Brookhaven National Laboratory (BNL) for irradiation. Upon arrival they were acclimated for at least 2 days prior to being irradiated with 0.1, 0.2, 0.4, or 1 Gy of 300 MeV/n ^28^Si ions (LET  =  64 keV/µm), 600 MeV/n ^56^Fe ions (LET  =  181 keV/µm) or 1 or 2 Gy of 1972SPE protons (30 MeV to 80 MeV) at the NASA Space Radiation Laboratory at BNL. Additional mice were irradiated at BNL with ^137^Cs gamma rays at doses of 1, 2, or 3 Gy. Numbers of animals used were based on our previous study described above. Male mice were selected because they have a higher incidence of radiation-induced AML than female mice. Control mice were sham-irradiated at BNL under the same conditions as the irradiated groups. Because of the large numbers of animals in each group, each dose group consisted of two cohorts irradiated approximately 12 months apart. Doses selected were based on previous studies [9]. The mice were not anesthetized during irradiation or any part of the study. Details of the irradiation conditions have been described previously [9]. Briefly, mice were loaded headfirst into 50 ml polypropylene centrifuge tubes with 8–10 predrilled 1/8 inch holes in the bottom. Ten tubes were then placed into a foam holder affixed perpendicular to the beam trajectory. The proton beam was delivered at 6 different energies, from 30 MeV to 80 MeV, in 10 MeV increments. The total dose was made up of partial doses for each energy, the dominant one being the 30 MeV dose, which makes up more than 90% of the total. The dose distribution was determined by scaling down the depth-dose spectrum from an SPE based on several recorded ones, from that expected in a 30 cm diameter water cylinder (man) to a 3 cm cylinder (mouse). The energy distribution which would yield that particular depth-dose spectrum was approximated with the 6 said energy bins, and the dose for each was fixed. Six separate setup and calibration files were created for each energy, so the beam could be delivered to the target stand sequentially without moving the targets in and out of the beam for each change. Before reaching the target, the beam transverses a large ion chamber previously calibrated with a NIST-traceable calibration chamber. The large chamber is used to automatically cut the beam when the desired dose is reached. The range for the majority of the SPE1972 proton beam is less than 9 mm and the width of a mouse is just under 3 cm.

The numbers of animals in each dose group are shown in Table 1. Mice were shipped to the Animal Resource Center at the University of Texas Medical Branch (UTMB) 3 to 5 days post-irradiation. At UTMB animals were housed 5 per cage in ventilated cage racks using Harlan Sani-chip bedding and maintained on an Harlan 7912 diet and autoclaved reverse osmosis purified water *ad libitum* throughout the study.

**Table 1:** Incidences of AML and HCC as a function of dose and radiation quality.

Radiation Quality	Dose in cGy (Initial Animal Numbers)	Frequency of AML (incidence ± SE^a^)	Frequency of HCC (Incidence ± SE)
None	0 (300)	1/280 (0.36 ± 0.36)	46/280 (16.5 ± 2.5)
300 MeV/n ^28^Si	10 (300)	6/298 (2.0 ± 0.8)	124/298 (41.6 ± 4.7)
	20 (300)	10/290 (3.4 ± 1.1)	150/290 (51.7 ± 3.0)
	40 (200)	7/189 (3.7 ± 1.4)	82/189 (43.4 ± 3.6)
	100 (200)	8/185 (4.3 ± 2.1)	78/185 (42.2 ± 3.6)
600 MeV/n ^56^Fe	10 (300)	5/288 (1.7 ± 0.8)	109/288 (37.8 ± 4.6)
	20 (300)	13/295 (4.4 ± 1.7)	143/295 (48.4 ± 2.9)
	40 (200)	1/189 (0.5 ± 0.5)	83/189 (43.9 ± 2.9)
	100 (200)	3/180 (1.6 ± 0.9)	74/180 (41.7 ± 3.7)
^137^Cs Gamma Rays	100 (400)	10/386 (2.6 ± 0.8)	54/386 14.0 ± 1.8
	200 (300)	19/276 (6.9 ± 1.5)	47/276 (17.0 ± 2.3)
	300 (100)	15/98 (15.3 ± 3.9)	23/98 (23.0 ± 4.3)
1972SPE Protons	100 (400)	10/392 (2.6 ± 0.8)	51/392 (13.0 ± 1.7)
	200 (300)	17/287 (5.9 ± 1.4)	49/287 (17.1 ± 2.2)

a As calculated assuming binomial distribution.

Animals were monitored daily and palpated weekly. They were euthanized when moribund and/or displaying signs and symptoms of AML, HCC or other neoplasms or when they reached 800 days of age. Dead, moribund, or symptomatic animals were necropsied and tissues (lung, liver, spleen, other tumors or pathologies) taken for histological evaluation. All necropsies and histological examinations were performed blinded. The large majority of animals were necropsied promptly enough to allow collection of tissues suitable for evaluation and diagnosis. AML were diagnosed using H&E stained tissue sections in addition to immunohistochemistry for myeloperoxidase using an anti-myeloperoxidase antibody at a concentration of 1:200 (Thermo Scientific catalogue number RB-373-A). Liver tumors were classified as adenomas or adenocarcinomas [16] other types of liver tumors were seen occasionally but at a low frequency, and there was no apparent relationship between these tumors and radiation dose.

### Ethics Statement

All animal work was approved by the Institutional Animal Care and Use Committees at UTMB and BNL (OAW Animal Welfare Assurance Numbers A3314-01 and A3106-01) under protocols 0906043 and 393 respectively. Both facilities are AAALAC accredited. Euthanasia was by carbon dioxide inhalation.

### Statistical Analyses

For evaluation of the proportion of animals contracting either AML or HCC as a function of dose and radiation type a multinomial logistic regression was used and odds ratios calculated to test for significant differences in risks among the radiation and control groups and between the various radiation types using the chi-square test. The relative biological effectiveness (RBE) was calculated using a weighted linear regression model to determine the linear slopes for the ^28^Si ion, ^56^Fe ion, 1972SPE proton, and gamma ray dose responses. Linear slopes (y) of the gamma ray and 1972SPE proton AML and HCC dose responses were determined over the dose range of 0 to 2 Gy compared with 0 to 1 Gy for the ^28^Si ion and ^56^Fe ion AML dose responses, and 0 to 0.2 Gy for the ^28^Si ion and ^56^Fe ion HCC dose responses. The ratios of these slopes were used to determine the RBE values (Z  =  x/y).

For determining significance of differences in the frequency of metastasis between controls and radiation groups and between radiation types a chi-square test was used.

## Results

Although a variety of sporadic neoplasms were observed in irradiated and control animals, only AML and HCC were found to increase significantly as a function of radiation dose. The data with respect to dose response and RBE estimates for these two neoplasms are described below.

### Acute myeloid leukemia

**Figure 1:**
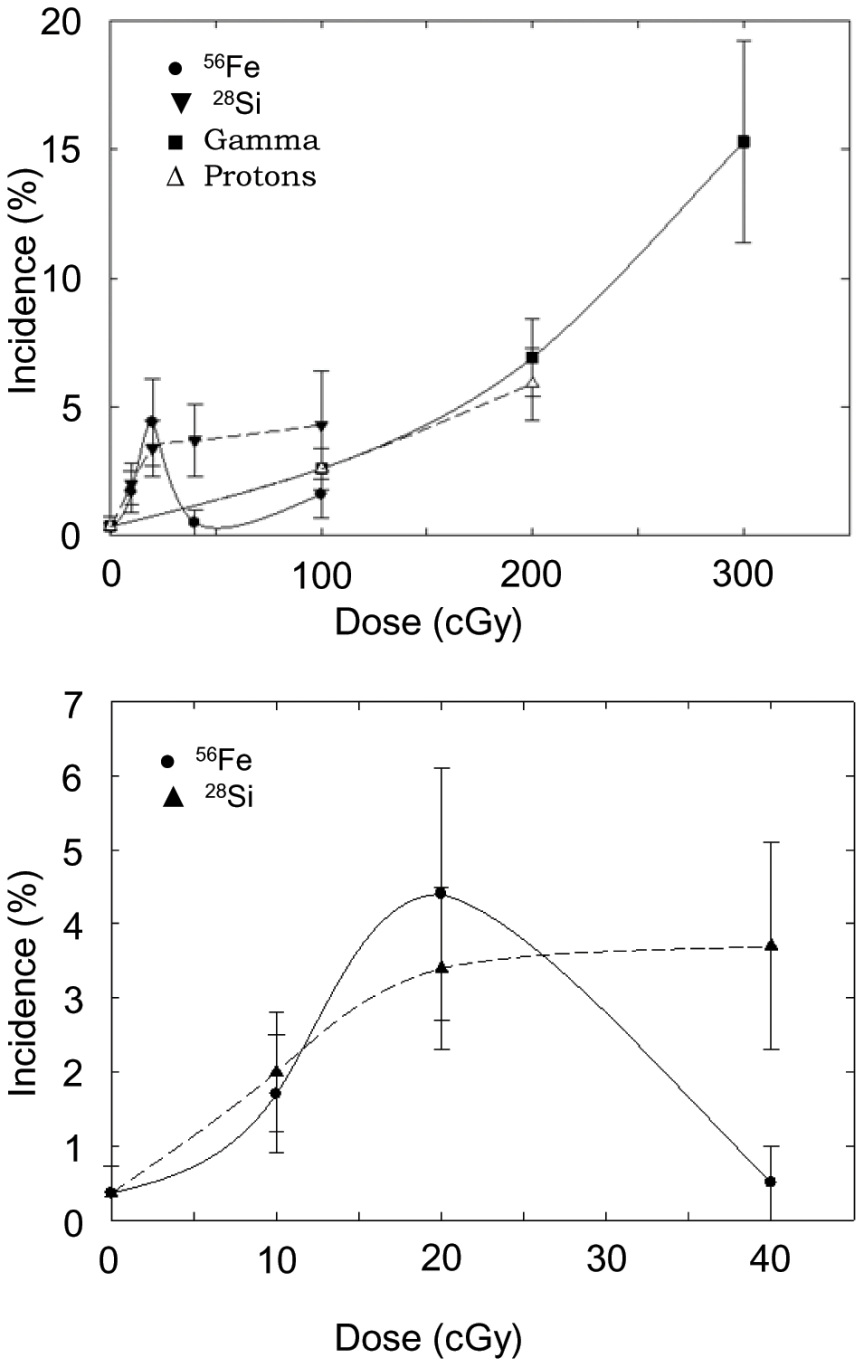
Incidence (%) of AML following exposure to 300 MeV/n ^28^Si (▾); 600 MeV/n ^56^Fe (•); ^137^Cs gamma rays (o); or 1972SPE protons (♦). The dose response over the 0-3 Gy dose range (top). The dose response over the 0–0.4 Gy dose range (bottom).

The incidences of AML as a function of dose and radiation type are shown in Table 1 and presented graphically in Figure 1. The time of appearance of these neoplasms ranged from 300 to 600 days with a median of 450–500 days of age. This range and median is similar to that previously reported previously for CBA mice [9], [17]. The dose response for the incidence of AML following exposure to gamma rays is also quite similar to that observed for both CBA and RFM mice [9], [18]. To determine whether there were significant differences between risks for induction of AML for the various radiation types, odds ratios were calculated and the significance of differences among groups was determined using the chi-square test. These analyses used the 0 to 2 Gy dose range for gamma rays and protons for comparison with the data over the 0 to 1 Gy dose range for the high LET radiations. Based on the results of these analyses over these dose ranges there were no significant differences between any of the radiation types.

The relative biological effectiveness (RBE) was calculated using a weighted linear regression model to determine the linear slopes for the ^28^Si ion, ^56^Fe ion, 1972SPE proton, and gamma ray dose responses. Linear slopes of the gamma ray and 1972SPE proton AML and HCC dose responses were determined over the dose range of 0 to 2 Gy compared with 0 to 1 Gy for the ^28^Si ion and ^56^Fe ion AML dose responses, and 0 to 0.2 Gy for the ^28^Si ion and ^56^Fe ion HCC dose responses. These data are shown in Tables 2 and 3. RBE values of approximately 1 or less were observed for 300 MeV/n ^28^Si ions, 600 MeV/n ^56^Fe, and 1972SPE protons when compared to ^137^Cs gamma ray exposures. These data are consistent with our previous studies examining AML induction by 1GeV/n ^56^Fe ions for which an RBE of essentially 1 was also obtained. It should be noted that there is an increase in the incidence of AML at the 0.2 Gy dose over that observed at other doses. This results in an apparent hump in the dose response. For ^56^Fe ions there was also a substantial decrease at 0.4 Gy. However, it is not appropriate to over interpret such detailed results in an observational study. This is especially the case in situations when the numbers of tumors in each dose group are relatively small and an increase or decrease of one or two tumors in any dose group can have a large impact on tumor incidence for that group. In this regard, we considered the possibility that the 0.2 Gy and 0.4 ^56^Fe ion results were outliers. However, for the sake of completeness multiple RBEs were determined for each HZE ion based on initial slope ratios calculated through 2 Gy for gamma rays and through 0.2, 0.4 or 1.0 Gy for the HZE ions (data not shown). The preponderance of the data supports an RBE of approximately 1, although it could be as high as 5 if the analyses were limited to data over 0 to 0.2 Gy. However, such an approach is not warranted based on the present data. To use this approach the current results would need to be confirmed in follow-up studies.

**Table 2:** Slopes determined using weighted linear regression for the induction of AML or HCC as a function of radiation quality^a^.

Cancer Type	Radiation	α	c	R^2^
AML	600 MeV/n ^56^Fe 0 to 1 Gy	0.008 ± 0.015	0.61 ± 0.71	0.083
	300 MeV/n ^28^Si 0 to 1 Gy	0.047 ± 0.017	0.71 ± 0.47	0.712
	γ-rays 0 to 2 Gy	0.037 ± 0.008	0.22 ± 0.56	0.910
	1972SPE Protons 0 to 2 Gy	0.026 ± 0.003	0.34 ± 0.15	0.989
HCC	600 MeV/n ^56^Fe 0 to 0.2 Gy	1.61 ± 0.31	18.2 ± 4.0	0.964
	300 MeV/n ^28^Si 0 to 0.2 Gy	1.77 ± 0.44	18.9 ± 5.6	0.943
	γ-rays 0 to 2 Gy	0.024 ± 0.014	14.1 ± 2.6	0.596
	1972SPE Protons 0 to 0.2 Gy	0.004± 0.022	15.2 ± 2.8	0.025

a Fit as I  =  αD + c.

**Table 3:** RBE values (± S.D) for 300 MeV/n ^28^Si, 600 MeV/n ^56^Fe, and 1972SPE protons.

Cancer Type	Radiation	RBE
AML 0–1 Gy dose range	600 MeV/n ^56^Fe	0.2 ± 0.4
	300 MeV/n ^28^Si	1.2 ± 0.5
	1972SPE Protons	0.7 ± 0.2
HCC 0–0.2 Gy dose range	600 MeV/n ^56^Fe	67 ± 41
	300 MeV/n ^28^Si	74 ± 47
	1972SPE Protons	1 ± 10

### Hepatocellular carcinoma

**Figure 2:**
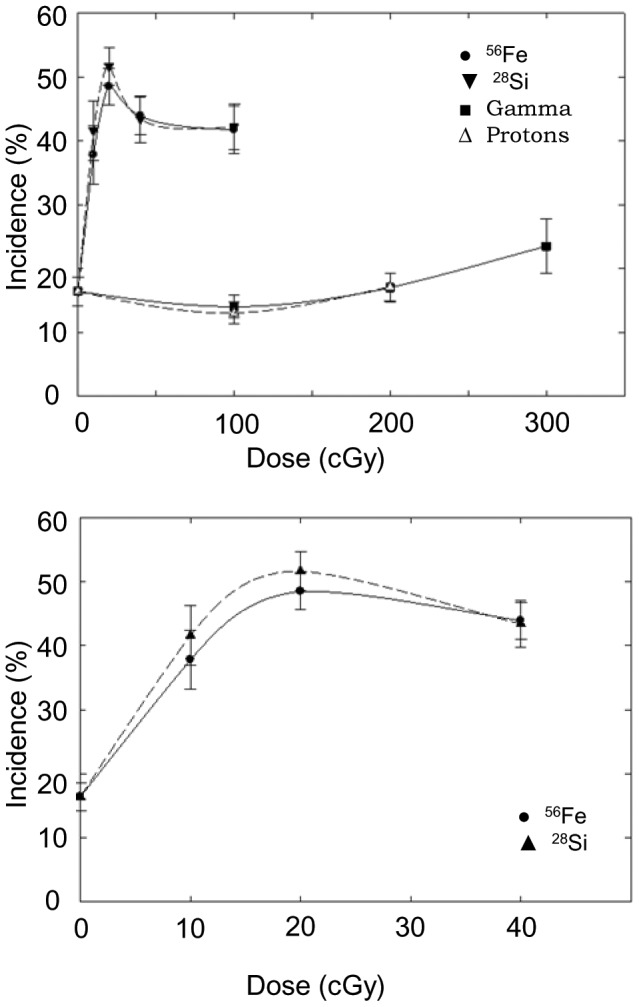
Incidence (%) of HCC following exposure to 300 MeV/n ^28^Si (▾); 600 MeV/n ^56^Fe (•); ^137^Cs gamma rays (o); or 1972SPE protons (♦). The dose response over the 0–3 Gy dose range (top). The dose response over the 0–0.4 Gy dose range (bottom).

The time of appearance of HCC was later than that for AML with a range of 300 to 800 days of age and a median of approximately 650 days for control and irradiated groups. This median time of appearance was not substantially different as a function of dose or radiation type. The incidence of HCC by dose and radiation type is shown in Table 1 and presented graphically in Figure 2. There was only a very slight increase in the incidence of HCC with increasing dose of gamma rays with the dose response for proton irradiated mice being similar. Analysis of these data based on chi-square analysis from calculated odds ratios as described in the methods section found no statistically significant increase in risks for gamma rays or protons compared to controls. Because of the steep dose response for HZE ion-induced HCC the calculations were limited to the 0.2 Gy dose range. As shown previously for 1 GeV/n ^56^Fe ions in CBA mice, there is a large, statistically significant increase in the risks of HCC over the 0 to 0.2 Gy dose range for both ^28^Si and ^56^Fe ions compared to gamma rays and protons over the 0 to 2 Gy dose range (p< 0.008 and 0.004 respectively) based on chi-square analysis from calculated odds ratios. RBE values derived from the slope ratios (Tables 2 and 3) in the range of 65 to 70 were obtained, with that for 300 MeV/n ^28^Si ions being slightly but not significantly higher than that for 600 MeV/n ^56^Fe ions. Importantly with respect to risk, while somewhat higher than the RBE reported for 1 GeV/n ^56^Fe ions, the data are consistent with previous results indicating a more convex shaped dose response curve with the maximum incidence in the 0.2 to 0.4 Gy dose range and very high RBE values following irradiation with HZE ions in this energy range.

### Metastatic HCC

**Figure 3:**
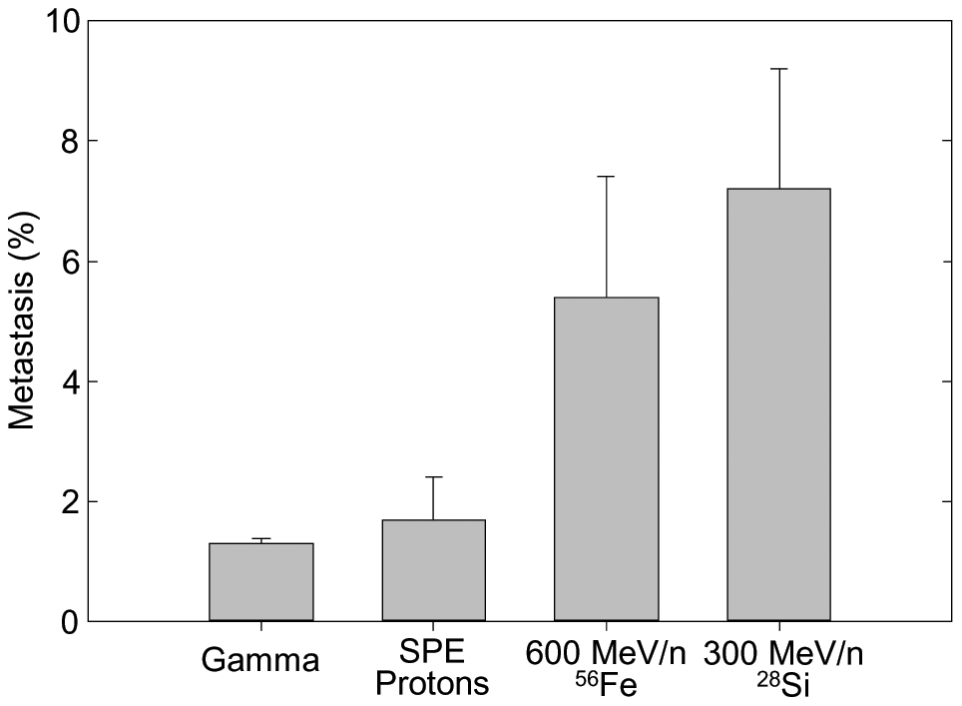
Incidence (%) of HCC with metastases to the lung as a function of radiation type.

During histological examination some of the HCC were found to have metastasized to the lung. Because of speculation about qualitative as well as quantitative differences between HZE ion and gamma ray induced tumors [19], we decided to determine whether there were differences in the frequency of metastases among the various radiation groups. Since the numbers of metastases were relatively low in any given dose group and the frequencies of these metastases were evenly distributed among dose groups with no apparent dose response relationship, we pooled the data and compared the overall frequencies of metastases as a function of radiation type. These data are shown in Figure 3. Based on chi-square analysis the frequency of lung metastases was significantly higher in both the ^28^Si and ^56^Fe ion groups when compared to gamma ray or proton irradiation (p<0.05). No significant differences in metastasis frequencies were found between spontaneous tumors and those arising in gamma ray and proton irradiated mice. It should be noted that lungs were not systematically examined for metastases. However, all apparent tumors in the lungs were taken for diagnosis. In those instances when the lung appeared to be tumor free by gross examination, the right lobe of the lung was systematically taken for examination for occult tumors. Therefore, these results are likely to underestimate the actual metastatic frequencies. Since necropsy technicians and the histopathologist were blinded to the radiation histories of the individual mice, it is unlikely that the data are biased with respect to any particular radiation type. Further, the frequencies of metastases for each radiation type were consistent in both irradiation cohorts which means that the results for metastatic frequencies were consistent in two groups of animals irradiated at different times. There was no apparent effect with respect to time of appearance of the primary liver tumor and the probability of metastasis. Therefore, we believe these data to be a true representation of increased metastatic frequency differences among groups.

## Discussion

This study has extended previous data for the induction of AML and HCC as a function of dose to include the effects of 300 MeV/n ^28^Si ions, 600 MeV/n ^56^Fe ions, and 1972SPE protons. The similar leukemogenic effects of gamma rays and 1972SPE protons in the present study are consistent with the finding by Clapp et al [20] that 60 MeV/n protons were just slightly less effective than 250 kVp X-rays for AML induction in RF mice. An RBE of 1 in this study over the dose range of 0–1 Gy for 300 MeV/n ^28^Si and 600 MeV/n ^56^Fe ions as compared to gamma rays was also consistent with our previous finding of an RBE of approximately 1 for 1 GeV/n ^56^Fe ions in CBA mice [9].

**Figure 4:**
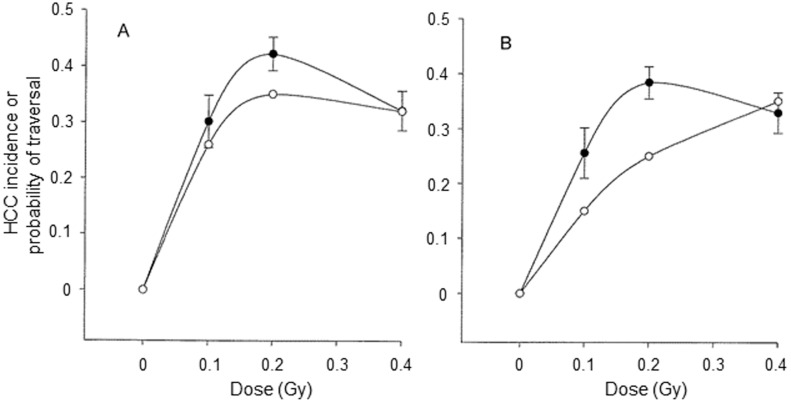
The probability for development of HCC corrected for background (solid line) with Abbott's correction [34] and the probability of a single traversal of a 50 µm^2^ hepatocyte nucleus (dashed line) with a 300 MeV/n ^28^Si ion (A) or 600 MeV/n ^56^Fe ion (B).

As with previous data for effects of 1 GeV/n ^56^Fe ions, we observed a steep dose response for the induction of HCC at low doses (0–0.2 Gy) of 300 MeV/n ^28^Si and 600 MeV/n ^56^Fe ions followed by a bend-over/plateau, i.e., a convex shaped dose response. While irradiation with 300 MeV/n ^28^Si ions or 600 MeV/n ^56^Fe ions resulted in a sharp increase in the incidence of HCC, irradiation with ^137^Cs gamma rays or 1972SPE protons had very little effect on incidence. Importantly, there was no significant difference between the effects of gamma rays and 1972SPE protons indicating an RBE of approximately 1 for 1972SPE protons. As a result of the substantial differences in the dose responses, calculated RBE values for 300 MeV/n ^28^Si and 600 MeV/n ^56^Fe ions were very high (in the range of 60–70). Such high values are consistent with our previous results for HCC following irradiation with 1 GeV/n ^56^Fe ions. Similar dose responses have also been reported for Harderian gland tumors in mice, mammary tumors in both mice and rats, and lung tumors in mice following irradiation with fission spectrum neutrons [21], [22]. The reason for this steep dose response with very high frequencies of HCC and other epithelial tumors at doses as low as 0.1 Gy (in some instance as low as 0.025 Gy) is a matter of speculation and is of considerable interest with respect to mechanisms and to evaluation of risks in humans. One factor which might play an important role in determining the form of the dose response after irradiation with HZE and which could be responsible for the apparent bend-over at doses in the range of 0.2 to 0.4 Gy is dose distribution alone. If a single hit were sufficient for cell transformation after irradiation with 300 MeV/n ^28^Si and with 600 MeV/n or 1 GeV/n ^56^Fe ions then a linear dose response followed by a plateau or bend-over for tumor induction would be predicted based on the probability of an hepatocyte being traversed as a function of dose. To examine this idea the relationship between the probability of an hepatocyte being traversed by either a ^28^Si ion or a ^56^Fe ion and the probability of HCC as a function of dose was examined and the data are shown in figures 4A and B. These data clearly demonstrate that at low doses the probability of a cell traversal is lower than the probability of the induction of HCC. Based on this it would appear that the dose response for the induction of HCC following exposure to HZE ions is more complex than simply a matter of dose distribution. This conclusion is supported by earlier microbeam studies on cellular effects following single cell irradiation *in vitro* [23], [24].

A more attractive hypothesis, which is supported by several lines of evidence, is that irradiation with HZE ions and other high LET radiations such as fission neutrons results in non-targeted effects (NTE) on neighboring non-irradiated cells involving changes in the microenvironment that serve to promote tumor development and progression.

With respect to tumor induction following high LET radiation exposure Cucinotta and Chappell [7] have modeled the dose response relationships for the induction of Harderian gland tumors following irradiations with a range of HZE ions over a broad spectrum of LETs comparing a linear model with a model factoring in NTE such as the release of clastogenic factors, the increased expression of cytokines and up-regulation of inflammatory pathways, and genomic instability. Their analysis demonstrated empirically that the NTE model provided a better fit to the dose response data, especially at low doses, than did a classical biophysical model for which an initial linear slope would be predicted. More direct support for such an NTE model and for effects on progression following high LET radiations come from animal studies of the carcinogenic processes involved in the development of mouse mammary cancer following exposure to fission spectrum neutrons [25]. In addition, Datta et al [19] have shown that heavy ion radiation exposure results in higher intestinal tumor frequency and greater β-catenin activation than gamma radiation in *Apc^Min/+^* mice. Further, Cheema et al [26] demonstrated that HZE ion exposure is more effective than an equitoxic dose of γ-ray radiation in increasing levels of metabolites indicative of persistent (2 month post-exposure) inflammation.

Beyond radiation-induced cancer the role of the microenvironment has been shown to be a major factor in carcinogenic processes in general [27]. Of particular importance is up-regulation of inflammatory pathways and increased expression of cytokines. Such effects have the potential to increase the probability of initiation, the probability of progression of these initiated cells to the neoplastic phenotype, and the probability of metastasis. Of particular importance in this regard is chronic inflammation. Importantly, subclinical inflammation may be as important as frank inflammation in increasing cancer risks.

The role of inflammation in the development of liver cancer is well documented. In humans, inflammation is a primary driver of liver cancer and a common feature of three major causes of hepatocellular carcinoma: infection with hepatitis B virus or hepatitis C virus; alcoholic cirrhosis; and obesity [28]–[30]. Animal models of liver cancer development including mechanistic studies on the induction and progression of preneoplastic foci have made a major impact on the current understanding of the neoplastic process. Results from these animal models also support an important role for inflammatory processes in the pathogenesis of liver cancer.

The increased frequency of lung metastases supports the hypothesis that qualitative as well as quantitative differences exist for radiation effects on neoplastic processes following irradiation with the HZE ions used in this study. Such qualitative differences are consistent with the NTE model and would, in fact, be predicted by a number of studies suggesting that changes in the tissue microenvironment associated with up-regulation of cytokines and inflammatory pathways increases the probability of tumor progression [27]. Importantly, this NTE model appears to apply to tumor induction for the HZE ions used in this study but not for gamma ray or proton irradiation for which a more biophysically based model appears to apply.

The finding of increased metastases from HZE ion-induced HCC is important because current NASA radiation exposure limits for space-flight crew members are based on not exceeding a 3% risk of fatal cancer at the upper 95% confidence interval [10]. If HZE ion-induced cancers are more metastatic, and consequently more lethal, the incidence to mortality ratio for spontaneous tumors used to predict cancer deaths in the current NASA risk model may be inappropriate and the radiation exposures now permitted may be excessive [31].

As described above, the data reported here confirm a low HZE ion RBE for the induction of AML over a dose range of 0 to 1 Gy with a potential RBE of 3 to 5 over the 0 to 0.2 Gy dose range. The data also confirm a very high RBE for the induction of HCC over the 0 to 0.2 Gy dose range. The difference in RBE values for leukemogenesis compared with HCC is a matter of speculation. One possible explanation is related to apparent differences in the pathways involved and the degree to which target cell intrinsic effects as opposed to tissue microenvironment may contribute to the pathogenesis of AML versus solid tumors. It is thought that only a few steps are required for the development of murine AML and that it is principally driven by an initial deletion in chromosome 2 with the dose response for this alteration predominating with respect to the shape of the AML dose response. Because of this, the interplay between the induction of chromosome 2 deletions and killing of target cells is a major factor in determining dose response relationships and by definition RBE. It should be pointed out that this does not preclude an important role for the microenvironment in radiation leukemogenesis, particularly the hematopoietic niche. Evidence for the strong role played by chromosome 2 aberrations in murine AML comes from a study of Darakashan et al [32] who demonstrated that a primary factor in multiple strain differences (including C3H mice) in sensitivity to AML is sensitivity to chromosome 2 aberrations. How this may apply to risks in humans is not known, but what is known is that a large proportion of radiation-induced AML arising as second cancers in patients treated with radiotherapy involve chromosomal deletions in chromosomes 5 and/or 7 [33]. On the other hand, based on current data, the mechanistic pathways involved in the development of epithelial tumors appear to be much more complex with a larger role for the interplay of cellular alterations and changes in cell signaling, cell-cell interactions, and other effects on the microenvironment which are likely to act to promote the neoplastic process and the progression of altered cells toward the neoplastic phenotype. Several studies examining radiation-induced cancer provide strong direct evidence for such a model. The increase in numbers of metastases in the HZE ion-induced cancers in this study would also tend to support such an effect on tumor progression. Such effects may play a particularly important role at low doses at which only a small proportion of target cells are traversed.

Based on the above discussion, it might be predicted that a majority of epithelial cancers induced by high LET ions would have a similar NTE associated dose response and high RBE values. On the other hand tumors, both leukemias and epithelial cancers, for which early cellular events and cell killing play a predominant role in the neoplastic process would tend to have dose responses more compatible with biophysical models with lower RBE values when compared to gamma rays. Whether such predictions hold and what factors are involved in effects on progression in those tumors for which NTE play a major role will require additional systematic studies to test these hypotheses. Clearly these are important questions with respect to our ability to estimate human risks using model systems. Once such factors can be identified studies to determine if similar events take place in human tissues and organs would facilitate a more direct approach to risk assessment.
